# Irradiation of pediatric glioblastoma cells promotes radioresistance and enhances glioma malignancy *via* genome-wide transcriptome changes

**DOI:** 10.18632/oncotarget.26137

**Published:** 2018-09-25

**Authors:** Hisham S. Alhajala, Ha S. Nguyen, Saman Shabani, Benjamin Best, Mayank Kaushal, Mona M. Al-Gizawiy, Eun-Young Erin Ahn, Jeffery A. Knipstein, Shama Mirza, Kathleen M. Schmainda, Christopher R. Chitambar, Ninh B. Doan

**Affiliations:** ^1^ Department of Medicine, Hematology and Oncology, Medical College of Wisconsin, Milwaukee, WI, USA; ^2^ Department of Neurosurgery, Medical College of Wisconsin, Milwaukee, WI, USA; ^3^ Department of Radiology, Medical College of Wisconsin, Milwaukee, WI, USA; ^4^ Department of Pediatrics, Hematology and Oncology, Medical College of Wisconsin, Milwaukee, WI, USA; ^5^ Department of Biophysics, Medical College of Wisconsin, Milwaukee, WI, USA; ^6^ Department of Chemistry and Biochemistry, University of Wisconsin, Milwaukee, WI, USA; ^7^ Mitchell Cancer Institute, University of South Alabama, Mobile, AL, USA

**Keywords:** pediatric glioblastoma, high-grade glioma, radioresistance, mRNA, ribonucleotide reductase

## Abstract

Pediatric glioblastoma (GBM) is a relatively rare brain tumor in children that has a dismal prognosis. Surgery followed by radiotherapy is the main treatment protocol used for older patients. The benefit of adjuvant chemotherapy is still limited due to a poor understanding of the underlying molecular and genetic changes that occur with irradiation of the tumor. In this study, we performed total RNA sequencing on an established stable radioresistant pediatric GBM cell line to identify mRNA expression changes following radiation. The expression of many genes was altered in the radioresistant pediatric GBM model. These genes have never before been reported to be associated with the development of radioresistant GBM. In addition to exhibiting an accelerated growth rate, radioresistant GBM cells also have overexpression of the DNA synthesis-rate-limiting enzyme ribonucleotide reductase, and pro-cathepsin B. These newly identified genes should be concertedly studied to better understand their role in pediatric GBM recurrence and progression after radiation. It was observed that the changes in multiple biological pathways protected GBM cells against radiation and transformed them to a more malignant form. These changes emphasize the importance of developing a treatment regimen that consists of a multiple-agent cocktail that acts on multiple implicated pathways to effectively target irradiated pediatric GBM. An alternative to radiation or a novel therapy that targets differentially expressed genes, such as metalloproteases, growth factors, and oncogenes and aim to minimize oncogenic changes following radiation is necessary to improve recurrent GBM survival.

## INTRODUCTION

Pediatric glioblastoma (GBM) is a relatively rare primary brain tumor in children [1]. Maximum surgical resection is considered the key and prognosis-determining factor in the treatment, followed by radiotherapy [1, 2]. Unfortunately, pediatric GBM is poorly studied at the molecular and genomic levels. Radiotherapy plays a critical role in eradicating the post-surgical residual microtumor [3]. Yet the molecular and genomic changes post-radiation in pediatric GBM have not been well examined. We have recently identified many radiation-responsive genes in adult radioresistant GBM cells that explain the radioresistance and increased malignant features of recurrent GBM [4]. However, adult and pediatric GBMs are distinct from each other at both molecular and genetic levels [2]. We are presenting a full RNA sequencing profile of both the radiation-naïve pediatric GBM (SJ-GBM2) cell line and stable radioresistant pediatric GBM (SJ-GBM2-10gy) cell line that we recently developed [5, 6]. Our data demonstrated that radiation perturbed the expression of many genes related to many different known pathways in cancer biology. The irradiated cells exhibited an enhanced growth rate, overexpressed protease cathepsin B, and both subunits of the rate-limiting enzyme of DNA synthesis ribonucleotide reductase (RR). In this study, we shed light on the irradiation responsive mRNA changes that transform the tumor cells toward a more aggressive and resistant form, for which treatment choices are limited. This study opens the door to further examining the possibility of targeting these modified pathways as a therapeutic strategy to block GBM tumor recurrence and progression.

## RESULTS

### Radioresistant irradiated pediatric GBM cells exhibited higher growth rate than control cells

**Figure 1:**
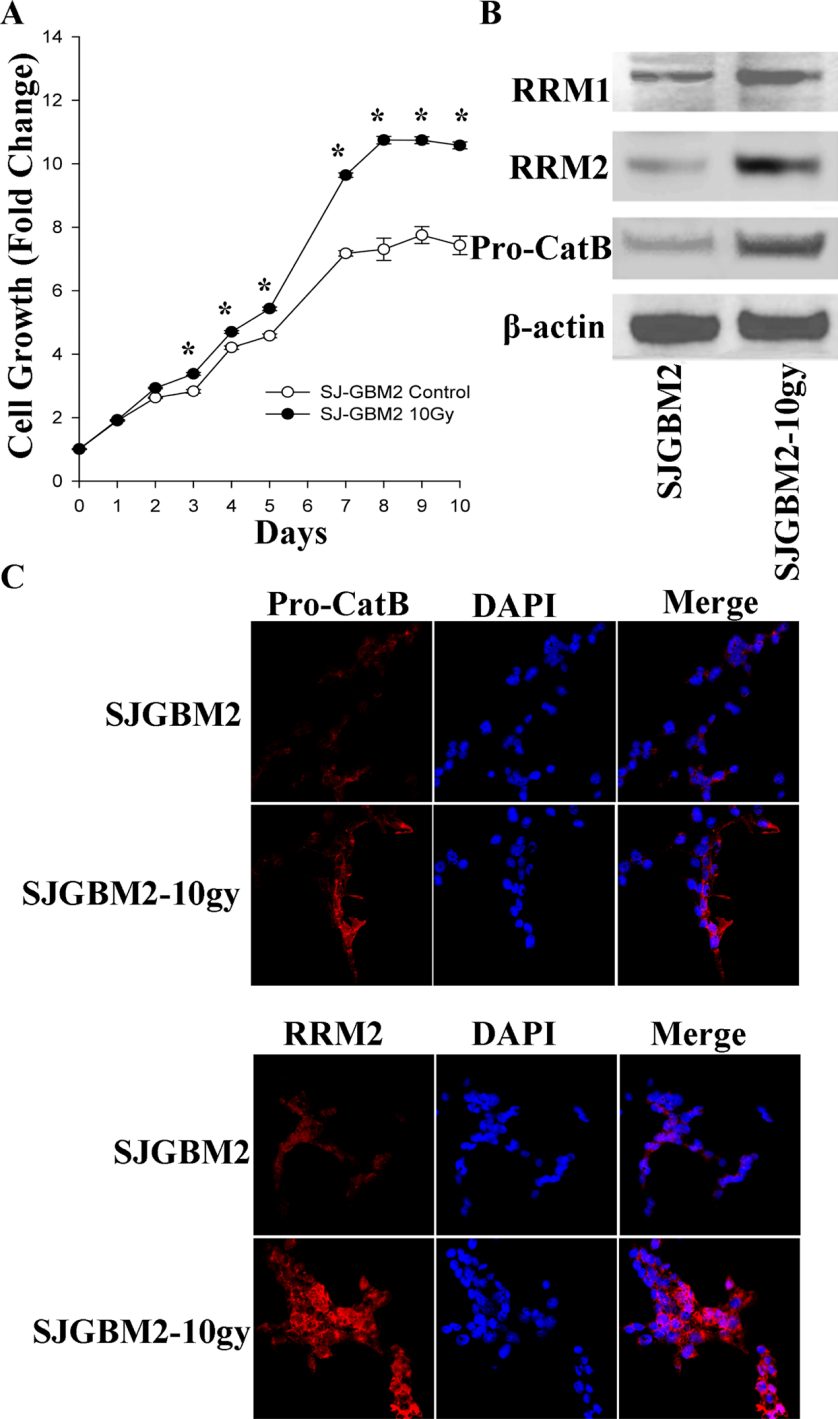
Irradiation of the pediatric GBM cells enhanced proliferation and expression of malignant-promoting proteins: (**A**) Growth curves of SJGBM2 and SJGBM2-10gy cells. (**B**) Western blot for RR M1 (94 kDa) and M2 (45 kDa) subunits and pro-cathepsin B (Pro-CatB) (43 kDa) in SJGBM2 and SJGBM2-10gy cells. (**C**) Direct immunofluorescence probing for RRM2 and cathepsin B (Pro-CatB) in both cells after a 24 h incubation in fresh medium. ^*^P < 0.05.

The *in-vitro* growth rate of the SJ-GBM2 and SJ-GBM2-10gy cells was evaluated using an MTT growth assay over a period of 10 days. SJ-GBM2-10gy cells showed a superior divergent growth starting from day 3, having the difference in growth maximized on day 7 when the control cells significantly slowed down their proliferation rate (Figure 1). The control cells SJ-GBM2 reached about 7.4 growth fold in 10 days, while the irradiated cells reached a 10.5 fold from their baseline. This represents an estimated 30% increase in growth (Figure 1A).

### Irradiated radioresistant pediatric GBM overexpresses ribonucleotide reductase

To probe for a mechanism promoting the superior growth rate in irradiated cells, the expression of both ribonucleotide reductase (RR) subunits was measured. The RR enzyme, specifically the RRM2 subunit, has been reported to be essential for proliferation and invasion of GBM cells [7]. Immunoblotting of control and irradiated cells revealed an increase in the expression of RRM1 subunit by 2-fold, and an increase in the RRM2 subunit by 3.5-fold in irradiated cells relative to control cells (Figure 1B). This increase in cellular expression of RRM2 was confirmed by immunofluorescence probing of intact cells, demonstrating the distribution of RRM2 in the cytoplasm of the irradiated cells and was greater than the control cells (Figure 1B, 1C).

### Irradiated radioresistant pediatric GBM overexpresses pro-cathepsin B

We were interested in evaluating whether protease may play of role in promoting invasion and progression of irradiated radioresistant GBM. Cathepsin B, a cysteine protease, has been shown to play a role in tumor growth and invasion [8, 9]. We probed for the differential expression level of pro-cathepsin B in control and irradiated cells. Western blot of cell lysates and immunofluorescence of intact cells revealed 3-fold overexpression of pro-cathepsin B in irradiated cells over control cells (Figure 1B, 1C). In addition to being localized to the cytoplasm, pro-cathepsin B was present in the processes of the irradiated cells, a feature that may be important in the invasiveness of GBM into the surrounding tissue (Figure 1C).

### Irradiation of pediatric GBM cells induces differential expression of 1192 radiation-responsive genes

Total mRNAs from SJGBM2 and SJGBM2-10gy cells were harvested and subjected to further analysis. To screen for global mRNA changes following irradiation, we profiled transcriptomes of the control SJ-GBM2 cell line and its derivative radioresistant irradiated SJ-GBM2-10gy cells by RNA sequencing (Table 1 and Supplementary Table 1). The criteria for differentially expressed genes were 2-fold or greater than statistically significant values (*P* < 0.05). We identified 1192 radiation responsive genes. Among these 1192 radiation responsive genes, 584 were upregulated and 608 were downregulated (Supplementary Tables 2 and 3).

**Table 1:** Enriched gene ontology categories of differentially expressed genes following irradiation based on sets of statistically significant (more than 2-fold) upregulated and downregulated genes (*P* < 0.05)

Differentially expressed	Category	*P*-value
**Upregulated**	GO:0048863: Stem cell differentiation	6.60E-03
	GO:0010628: Positive regulation of gene expression	7.70E-03
	GO:0002040: Sprouting angiogenesis	3.20E-02
	GO:0008284: Positive regulation of cells proliferation	2.40E-02
	GO:0070848: Response to growth factor	7.40E-02
	GO:0001558: Regulation of cell growth	7.60E-02
	GO:0055114: Oxidation reduction process	3.00E-02
	GO:0071356: Cellular response to tumor necrosis factor	3.60E-02
	GO:0006954: Inflammatory response	4.10E-02
	GO:0016055: Wnt signaling pathway	8.40E-02
	GO:0043066: Negative regulation of apoptotic process	9.40E-02
	GO:0004222: Metalloendopeptidase activity	2.40E-02
**Downregulated**	GO:0007155: Cell adhesion	4.90E-03
	GO:0043065: Positive regulation of apoptosis	1.30E-04
	GO:008285: Negative regulation of cell proliferation	4.10E-02
	GO:0002020: Protease binding	1.20E-03

Experiments were performed in triplicate.

### Upregulation of genes promoting tumor growth and aggressiveness following irradiation

Gene ontology analysis of the mRNA data was utilized to categorize genes into functional groups. It revealed that upregulated genes were enriched in positive regulation of stem cells differentiation, angiogenesis, cell proliferation, cell growth, inflammatory response, positive regulation of the Wnt signaling pathway, response to hypoxia, metalloendopeptidase activity, cellular response to tumor necrosis factor and negative regulation of apoptotic process (Table 1 and Supplementary Table 2, and Supplementary Table 4). The upregulated genes were enriched in positive regulation of gene expression and tumor cell proliferation such as KIT, connective tissue growth factor CTGF and ID1, ID2, and TLE1-FOXG1 transcriptional factors that have been reported to enhance growth and proliferation of GBM cells [10–13]. G protein-coupled receptor kinase 5 (GRK5) plays an important role in tumor cells’ proliferation [14]. Fibroblast growth factor 4 (FGF4) also has been correlated with a greater malignancy profile in high-grade gliomas [15].The radioresistant cells had upregulated expression of many anti-apoptotic genes, including BCL2, CD74, and WT1, which regulates GBM cells proliferation and apoptosis [16]. A significant upregulation (10-fold) of the AIM2 gene, a tumor-associated antigen, was found. This gene upregulation was observed in GBM patients and in malignant cell lines as well [17]. Proteolytic enzymes such as ADAM28, MMP12 and MMP17, which can enhance extracellular invasion and expansion of tumor volume, were also upregulated in the irradiated SJGBM2-10gy cells (Supplementary Table 4).

### Downregulated genes were enriched in the apoptotic process, tumor suppression, protease binding and cell adhesion

The gene ontology analysis was conducted to analyze differentially expressed genes. Compared with control cells, downregulated genes in the irradiated SJ-GBM2-10gy cells were enriched in growth inhibitor, transcription DNA-templated, cell adhesion, apoptotic process, and protease binding (Tables 1 and 2, Supplementary Table 3). Tumor irradiation has been shown to cause silencing of many tumor suppressor genes, which in turn enhances tumor aggressive growth and invasion [18]. Many of the well-known tumor suppressor genes were found downregulated in the irradiated SJGBM2-10gy including DAB2IP, ING2, interleukin 1 beta, and MEG3 (Table 2). Downregulation of DAB2IP induces radioresistance by accelerating DNA double strand repair after radiation and evasion of apoptotic process in prostate carcinoma [19]. Inhibitor of Growth Family member 2 (ING2) is a tumor suppressor that is involved in activation of p53/Tp53-dependent apoptosis [20]. Interleukin 1 beta is known to suppress GBM cells through promoting hypoxia-induced apoptosis by downregulation of HIF1 [21]. Maternally expressed gene 3 (MEG3) plays a role in activation of p53-dependent apoptosis and is found downregulated in the glioma cells compared with normal brain cells [22]. Interestingly, the irradiated cells showed significant repression of the main cellular metalloprotease inhibitor proteins called TIPM4 and alpha2 macroglobulin (A2M) [23–26]. Downregulation of protease inhibitors causes unopposed digestion of the extracellular matrix around the tumor by metalloproteases, which were also upregulated in irradiated cells, and therefore, facilitating tumor invasion of the extracellular space.

**Table 2:** Downregulated genes of selected enriched gene ontology categories following irradiation of SJ-GBM2 cells are shown based on sets of statistically significant changes (*P* < 0.05)

Gene ontology group	*P*-value	Fold changes	Gene symbol	Gene description
Cell adhesion	4.90E-03	0.3390	CD33	CD33 molecule
		0.4851	EDIL3	EGF like repeats and discoidin domains 3
		0.3426	EPHA4	EPH receptor A4
		0.4796	F11R	F11 receptor
		0.0456	L1CAM	L1 cell adhesion molecule
		0.3634	SPOCK1	SPARC/osteonectin, cwcv and kazal like domains proteoglycan 1
		0.1247	TNFAIP6	TNF alpha induced protein 6
		0.3202	ALCAM	activated leukocyte cell adhesion molecule
		0.4053	ADGRB1	adhesion G protein-coupled receptor B1
		0.4680	BCAM	basal cell adhesion molecule (Lutheran blood group)
		0.1871	CDH3	cadherin 3
		0.3202	CDH8	cadherin 8
		0.4662	COL5A1	collagen type V alpha 1 chain
		0.2467	COL8A1	collagen type VIII alpha 1 chain
		0.0839	CNTN2	contactin 2
		0.1860	CNTN4	contactin 4
		0.4905	CNTNAP3	contactin associated protein-like 3
		0.4111	EMP2	epithelial membrane protein 2
		0.2666	FAP	fibroblast activation protein alpha
		0.3239	LGALS3BP	galectin 3 binding protein
		0.3272	ITGA11	integrin subunit alpha 11
		0.3245	ITGA9	integrin subunit alpha 9
		0.2359	LAMA3	laminin subunit alpha 3
		0.2795	PTPRK	protein tyrosine phosphatase, receptor type K
		0.1867	ROBO2	roundabout guidance receptor 2
		0.2710	TNC	tenascin C
Positive regulation of apoptotic process	1.30E-04	0.4109	AKAP13	A-kinase anchoring protein 13
		0.3966	DAB2IP	DAB2 interacting protein
		0.3180	FGD4	FYVE, RhoGEF and PH domain containing 4
		0.3340	KLF11	Kruppel like factor 11
		0.2253	ARHGEF16	Rho guanine nucleotide exchange factor 16
		0.1708	ARHGEF4	Rho guanine nucleotide exchange factor 4
		0.3533	TNFRSF8	TNF receptor superfamily member 8
		0.4738	ALDH1A2	aldehyde dehydrogenase 1 family member A2
		0.1373	EEF1A2	eukaryotic translation elongation factor 1 alpha 2
		0.4137	GAL	galanin and GMAP prepropeptide
		0.3390	ING2	inhibitor of growth family member 2
		0.2705	IFIT2	interferon induced protein with tetratricopeptide repeats 2
		0.4339	IRF5	interferon regulatory factor 5
		0.4589	JMY	junction mediating and regulatory protein, p53 cofactor
		0.0767	NGFR	nerve growth factor receptor
		0.0816	PNMA2	paraneoplastic Ma antigen 2
		0.3719	PAWR	pro-apoptotic WT1 regulator
		0.4911	STK3	serine/threonine kinase 3
Negative regulation of cell proliferation	4.10E-02	0.2946	CEBPA	CCAAT/enhancer binding protein alpha
		0.3390	CD33	CD33 molecule
		0.3966	DAB2IP	DAB2 interacting protein
		0.4179	DLC1	DLC1 Rho GTPase activating protein
		0.3340	KLF11	Kruppel like factor 11
		0.2957	RERG	RAS like estrogen regulated growth inhibitor
		0.3533	TNFRSF8	TNF receptor superfamily member 8
		0.4053	ADGRB1	adhesion G protein-coupled receptor B1
		0.4738	ALDH1A2	aldehyde dehydrogenase 1 family member A2
		0.4351	CHD5	chromodomain helicase DNA binding protein 5
		0.1004	F2R	coagulation factor II thrombin receptor
		0.3390	ING2	inhibitor of growth family member 2
		0.4605	IRF1	interferon regulatory factor 1
		0.4571	IL1B	interleukin 1 beta
		0.3036	LDOC1	leucine zipper down-regulated in cancer 1
		0.4785	LIF	leukemia inhibitory factor
		0.0953	MEG3	maternally expressed 3 (non-protein coding)
		0.2795	PTPRK	protein tyrosine phosphatase, receptor type K
		0.4911	STK3	serine/threonine kinase 3
		0.4718	SLIT3	slit guidance ligand 3
Protease binding	1.20E-03	0.0331	CD70	CD70 molecule
		0.1539	TIMP4	TIMP metallopeptidase inhibitor 4
		0.2020	A2M	alpha-2-macroglobulin
		0.2780	CSTA	cystatin A
		0.1772	CST6	cystatin E/M
		0.2666	FAP	fibroblast activation protein alpha
		0.4143	IL1R1	interleukin 1 receptor type 1
		0.1026	RYR1	ryanodine receptor 1
		0.2443	SERPINA1	serpin family A member 1
		0.3182	SERPINA5	serpin family A member 5
		0.4862	SERPINE1	serpin family E member 1

Experiments were performed in triplicate.

## DISCUSSION

Tumor radiation plays a critical role in pediatric brain tumors after gross total resection [1–3]. Unlike brain tumors in adults, the benefit of adjuvant chemotherapy is limited. As a result, it is typical that solely radiotherapy is utilized to eradicate any remnant tumor cells after surgery [1–3]. Ironically, irradiation of tumors is known to transform tumor cells into a more aggressive, radioresistant form, for which the treatment options are limited [27]. However, post-radiation transcriptome changes of the pediatric GBM have not been closely studied. The pediatric GBM cell line SJ-GBM2 is a common line that has been used for *in vitro* studies and is considered a radiation-naive cell line as it has not previously been irradiated [28, 29]. We previously described a stable radioresistant pediatric GBM model of irradiated SJ-GBM2 cells [5, 30]. In the current study, we further characterized these radioresistant cells and examined mRNA changes induced by irradiation. The results showed that the irradiated cells were more aggressive and possessed a higher proliferation rate when compared with their progenitors. SJ-GBM2 cells showed 7.4-fold growth on day 10 of incubation, while the irradiated SJ-GBM2-10gy cells grew up to 10.5-fold during the same period, suggesting that irradiation promotes a higher cell proliferation rate in radioresitant cells (Figure 1A). This rapid growth was paralleled by the increase in the expression of RR subunits, the main enzymes involved in DNA synthesis during cell division (Figure 1B, 1C). RR activity is critical for tumor cell growth [31]. The RRM2 subunit specifically has been linked to DNA repair capacity after radiation [32]. Our data suggested that RR overexpression in the irradiated cells may contribute to their ability to grow after radiation. In order to understand the changes in gene expression induced by irradiation, we performed a complete RNA sequencing of SJ-GBM2 and SJ-GBM2-10gy cells. Of the 32998 genes sequenced, a relatively small number (3.6%) of genes following irradiation were differentially expressed by meeting the criteria of a more than two-fold change (Supplementary Table 1). The upregulated genes such as *KIT, ID1, ID2, GRK5, CTGF, LEF1*, *NTRK3* and *PGF* are considered oncogenes that promote tumor proliferation, invasion and gene expression, in GBM or other types of cancers [11, 12, 14, 33–36]. *KIT* is an oncogene that enhances GBM proliferation and growth and it was found to be upregulated in patient-derived GBM samples [10]. *BMP1* is involved in many signaling pathways in GBM and correlate with poor prognosis in glioma patient [37]. Among the upregulated genes there was a significant increase in the expression of glutathione peroxidase 3 (*GPX3*), which is known to be an oxidative stress-induced antioxidant [38]. This increase of *GPX3* may protect cells against reactive oxygen species produced after radiation. Gene ontology showed that many anti-apoptotic and anti-inflammatory genes were upregulated in the irradiated cells (Table 1 and Supplementary Table 4).

The downregulated genes were enriched in adhesion molecules (Table 2). High-grade gliomas alter their expression of extracellular matrix adhesion proteins for tumor progression and invasion into the normal surrounding brain tissue [39]. Irradiation of cells depressed the expression of many adhesion molecules such as *CHD3, CHD8, FAP, BCAM, L1CAM, TNC, ITGA11*, and *ITGA9*.

Eighteen pro-apoptotic genes, along with 21 tumor suppressor genes, were downregulated following irradiation (Table 2). This explains the acquired radioresistant feature of irradiated cells and the increased malignant nature that was observed in irradiated cells (Figure 1A). Specifically, the *DAB2IP* gene was found to play a tumor suppressor role in medulloblastoma and a lower expression of *DAB2IP* caused resistance to radiation in prostate carcinoma [19, 40]. *P53* is believed to be the regulator of many radiation responsive genes that alter tumor cells sensitivity to radiation [41]. Any loss or mutation in the *P53* function leads to radioresistance [41]. Our results revealed that the downregulation of genes involved in radiation responsive activation of *P53*-apoptotic pathway such as *ING2, IL2B* and *MEG3*, enabled cells to acquire radioresistance. In addition, irradiation also altered metalloprotease activity. Although irradiated cells have lower expression of protease inhibitors *TIPM4* and A2M, the expression of metalloproteases such as *MMP12, MMP17* and cathepsin B was augmented. The net effect is the enhancement in metalloprotease activity, allowing the invasion and expansion of GBM after radiation. Alpha-2 macroglobulin (A2M), a large plasma glycoprotein natural inhibitor of cathepsin B and many other growth factors especially epidermal growth factors, exhibits the capability of abrogating the malignant potential of astrocytoma cells such as cell proliferation, invasion, and migration, and, as such, it can be exploited for therapeutic purposes [25, 26, 42–46]. Alpha-2 macroglublin has been proposed as a molecule conferring cancer resistance to the long-lived (30-year lifespan) naked mole rat, whose A2M transcript level is 140-fold higher than that of the control, and no tumor has ever been observed in these rats [47–49].

In this study, we report the alterations of gene expressions in response to radiation, which might play a critical role in the acquisition of radioresistance by irradiated pediatric GBM cells. The results indicated that irradiated cells were not only radioresistant, but they also transformed into a more aggressive, malignant GBM. This parallels the clinical observation that recurrent GBM is more aggressive and malignant after irradiation. This malignant transformation emphasizes the importance of developing a treatment regimen consisting of a multiple-agent cocktail that acts on multiple implicated pathways to effectively target irradiated pediatric GBM. An alternative to radiation or perhaps a new therapy- targeting differentially expressed genes, such as metalloproteases, growth factors, and oncogenes- that aims to minimize oncogenic changes following radiation is necessary to improve recurrent pediatric GBM survival. As a main cellular inhibitor of all tumor-associated metalloproteinases, and a sequester of many growth factors, alpha 2-macroglobulin might be exploited as a novel therapy to modulate pediatric GBM growth and invasion [42–46].

## MATERIALS AND METHODS

### Reagents and cells

The pediatric glioblastoma cell line (SJ-GBM2) was obtained from the Children’s Oncology Group (COG), (Dallas, TX, USA). Cell lines were cultured in Eagle’s minimum essential medium containing 10% (v/v) fetal bovine serum, and supplemented with 1% sodium pyruvate and 0.1% gentamicin. Culture medium materials were obtained from Life Technologies, Inc. (Grand Island, NY, USA).

### Generation of the stable pedicatric radioresistant GBM model

We previously generated and described a stable radioresistant GBM model [5]. Briefly, to generate the SJ-GBM2-10gy, SJ-GBM2 cell lines were grown to confluence and then irradiated with a Pantak HF320 X-ray machine (Agfa NDT Ltd., Reading, UK) operating at 300 kV at a dosage of 2.09 Gy/min to a total radiation dose of 10 Gy. Over the course of weeks, most cells died and less than ∼1% of cells survived the irradiation. These radioresistant SJGBM2-10gy cells were allowed to grow to confluence and were perpetuated for experiments.

### MTT viability and Western blot assays

SJ-GBM2 and SJ-GBM2-10gy cell growths were measured by MTT assay. Cells were plated in an initial density of 0.05 × 10⁶ cell/ml in 96-well micro-well plates and incubated for 1 to 10 days. A daily readout of the cellular viability was recorded for growth rate measurement.

Cell lysates from both irradiated and control cell lines were analyzed for the expression of ribonucleotide reductase subunits and cathepsin B by Western blot. Antibodies used were mouse anti RRM1 (Santa Cruz Biotechnology, sc-377415), mouse anti-RRM2 (Santa Cruz Biotechnology, sc-376973), mouse anti pro-cathepsin B (Life Technologies, Inc., 414800) and mouse anti b-actin (Santa Cruz Biotechnology, sc-47778). Secondary antibody used was HRP goat anti mouse (Li-Cor, 926-80010).

### Immunofluorescence

SJ-GBM2 and SJ-GBM2-10gy cells were grown on chamber slides for 24 h. Cells were fixed with 3.7% paraformaldehyde in PBS, washed with ice-cold phosphate-buffered saline (PBS), permeabilized with 0.2% Triton 100 and incubated in 1% bovine serum albumin (BSA) in PBS blocking buffer. Next, cells then were incubated in 37° C with anti-pro-cathepsin B and anti-RRM2 for 1 h. Cells then were washed with 0.01% Tween PBS, and incubated in the dark with correspondent Texas horse anti mouse (Vector, TI2000) secondary antibodies for 1 h. Cells then were washed and counterstained with DAPI (Invitrogen, D1306) for 5 min.

Confocal images were captured with a Carl Zeiss LSM510 microscope (Jena, Germany) equipped with a plan-apochromat 20×/0.8 NA or 40×/1.2NA lens available at the Imaging Core Facility at the Children’s Research Institute, Medical College of Wisconsin (Milwaukee, WI, USA). Red fluorophore (A546) was excited with a diode laser (561 nm) and DAPI was excited with a 405 laser. Laser power to the sample was controlled with an acousto-optic tunable filter (AOTF) and the appropriate dichroics and filters for each fluorophore were used during image acquisition. The images were corrected for pixel saturation using with photomultiplier detector gain and amplifier offset controls as per the manufacturer’s recommendations. Images were taken four to eight times and collected using Aim 4.2 software.

### RNA library preparation and sequencing

RNA sequencing was done as previously described [4]. In brief, RNA-sequencing libraries were prepared using the TruSeq Stranded mRNA Library Prep Kit (Illumina, Inc., San Diego, CA, USA) according to the manufacturer’s protocol. PolyA mRNA from an input of 500 hg high quality total RNA (RINe > 8) was purified and fragmented. First strand complementary deoxyribonucleic acid (cDNA) syntheses were performed using random hexameres and ProtoScript II Reverse Transcriptase (New England BioLabs Inc., Ipswich, MA, USA). The 3′ ends of the cDNA were adenylated and then indexing adaptors were ligated. Polymerase chain reactions were used to selectively enrich those DNA fragments that have adapter molecules on both ends and to amplify the amount of DNA in the library.

The libraries were quantified using the Promega QuantiFluor dsDNA System on a Quantus Fluorometer (Promega, Madison, WI, USA). The size and purity of the libraries were analyzed using the High Sensitivity D1000 Screen Tape on an Agilent 2200 TapeStation instrument. The libraries were normalized, pooled, and subjected to cluster, and pair read sequencing was performed for 150 cycles on a HiSeq4000 instrument (Illumina, Inc., San Diego, CA, USA), according to the manufacturer’s instructions.

### Gene ontology analysis

The gene ontology enrichment analysis was performed using DAVID Bioinformatics Resources 6.7, NIAIS/NIH (http://david.abcc.ncifcrf.gov/).

## SUPPLEMENTARY MATERIALS TABLES











## Abbreviations

GBM: Giloblastoma
PBS: phosphate-buffered
SJ-GBM2: radiation-naïve pediatric GBM
RR: ribonucleotide reductase
SJ-GBM2-10gy: stable radioresistant pediatric GBM

## References

[1] Fangusaro J (2009). Pediatric high-grade gliomas and diffuse intrinsic pontine gliomas. J Child Neurol.

[2] Jones C, Perryman L, Hargrave D (2012). Paediatric and adult malignant glioma: close relatives or distant cousins?. Nat Rev Clin Oncol.

[3] Fallai C, Olmi P (1997). Hyperfractionated and accelerated radiation therapy in central nervous system tumors (malignant gliomas, pediatric tumors, and brain metastases). Radiother Oncol.

[4] Doan NB, Nguyen HS, Alhajala HS, Jaber B, Al-Gizawiy MM, Ahn EE, Mueller WM, Chitambar CR, Mirza SP, Schmainda KM (2018). Identification of radiation responsive genes and transcriptome profiling via complete RNA sequencing in a stable radioresistant U87 glioblastoma model. Oncotarget.

[5] Doan NB, Nguyen HS, Al-Gizawiy MM, Mueller WM, Sabbadini RA, Rand SD, Connelly JM, Chitambar CR, Schmainda KM, Mirza SP (2017). Acid ceramidase confers radioresistance to glioblastoma cells. Oncol Rep.

[6] Nguyen HS, Awad AJ, Shabani S, Doan N (2018). Molecular Targeting of Acid Ceramidase in Glioblastoma: A Review of Its Role, Potential Treatment, and Challenges. Pharmaceutics.

[7] Chitambar CR, Al-Gizawiy MM, Alhajala HS, Pechman KR, Wereley JP, Wujek R, Clark PA, Kuo JS, Antholine WE, Schmainda KM (2018). Gallium Maltolate Disrupts Tumor Iron Metabolism and Retards the Growth of Glioblastoma by Inhibiting Mitochondrial Function and Ribonucleotide Reductase. Mol Cancer Ther.

[8] Strojnik T, Kos J, Zidanik B, Golouh R, Lah T (1999). Cathepsin B immunohistochemical staining in tumor and endothelial cells is a new prognostic factor for survival in patients with brain tumors. Clin Cancer Res.

[9] Rempel SA, Rosenblum ML, Mikkelsen T, Yan PS, Ellis KD, Golembieski WA, Sameni M, Rozhin J, Ziegler G, Sloane BF (1994). Cathepsin B expression and localization in glioma progression and invasion. Cancer Res.

[10] Joensuu H, Puputti M, Sihto H, Tynninen O, Nupponen NN (2005). Amplification of genes encoding KIT, PDGFRalpha and VEGFR2 receptor tyrosine kinases is frequent in glioblastoma multiforme. J Pathol.

[11] Fong S, Debs RJ, Desprez PY (2004). Id genes and proteins as promising targets in cancer therapy. Trends Mol Med.

[12] Perk J, Iavarone A, Benezra R (2005). Id family of helix-loop-helix proteins in cancer. Nat Rev Cancer.

[13] Dali R, Verginelli F, Pramatarova A, Sladek R, Stifani S (2018). Characterization of a FOXG1:TLE1 transcriptional network in glioblastoma-initiating cells. Mol Oncol.

[14] Kim JI, Chakraborty P, Wang Z, Daaka Y (2012). G-protein coupled receptor kinase 5 regulates prostate tumor growth. J Urol.

[15] Phillips HS, Kharbanda S, Chen R, Forrest WF, Soriano RH, Wu TD, Misra A, Nigro JM, Colman H, Soroceanu L, Williams PM, Modrusan Z, Feuerstein BG (2006). Molecular subclasses of high-grade glioma predict prognosis, delineate a pattern of disease progression, and resemble stages in neurogenesis. Cancer Cell.

[16] Kijima N, Hashimoto N, Chiba Y, Fujimoto Y, Sugiyama H, Yoshimine T, van den Heuvel-Eibrink MM (2016). Functional Roles of Wilms’ Tumor 1 (WT1) in Malignant Brain Tumors.

[17] Liu G, Yu JS, Zeng G, Yin D, Xie D, Black KL, Ying H (2004). AIM-2: a novel tumor antigen is expressed and presented by human glioma cells. J Immunother.

[18] Ma H, Rao L, Wang HL, Mao ZW, Lei RH, Yang ZY, Qing H, Deng YL (2013). Transcriptome analysis of glioma cells for the dynamic response to γ-irradiation and dual regulation of apoptosis genes: a new insight into radiotherapy for glioblastomas. Cell Death Dis.

[19] Kong Z, Xie D, Boike T, Raghavan P, Burma S, Chen DJ, Habib AA, Chakraborty A, Hsieh JT, Saha D (2010). Downregulation of human DAB2IP gene expression in prostate cancer cells results in resistance to ionizing radiation. Cancer Res.

[20] Pedeux R, Sengupta S, Shen JC, Demidov ON, Saito S, Onogi H, Kumamoto K, Wincovitch S, Garfield SH, McMenamin M, Nagashima M, Grossman SR, Appella E (2005). ING2 regulates the onset of replicative senescence by induction of p300-dependent p53 acetylation. Mol Cell Biol.

[21] Sun W, Depping R, Jelkmann W (2014). Interleukin-1β promotes hypoxia-induced apoptosis of glioblastoma cells by inhibiting hypoxia-inducible factor-1 mediated adrenomedullin production. Cell Death Dis.

[22] Wang P, Ren Z, Sun P (2012). Overexpression of the long non-coding RNA MEG3 impairs *in vitro* glioma cell proliferation. J Cell Biochem.

[23] Brew K, Dinakarpandian D, Nagase H (2000). Tissue inhibitors of metalloproteinases: evolution, structure and function. Biochim Biophys Acta.

[24] Cuéllar JM, Cuéllar VG, Scuderi GJ (2016). α2-Macroglobulin: Autologous Protease Inhibition Technology. Phys Med Rehabil Clin N Am.

[25] Doan N, Gettins PG (2008). alpha-Macroglobulins are present in some gram-negative bacteria: characterization of the alpha2-macroglobulin from Escherichia coli. J Biol Chem.

[26] Doan N, Gettins PG (2007). Human alpha2-macroglobulin is composed of multiple domains, as predicted by homology with complement component C3. Biochem J.

[27] Wild-Bode C, Weller M, Rimner A, Dichgans J, Wick W (2001). Sublethal irradiation promotes migration and invasiveness of glioma cells: implications for radiotherapy of human glioblastoma. Cancer Res.

[28] Kang MH, Smith MA, Morton CL, Keshelava N, Houghton PJ, Reynolds CP (2011). National Cancer Institute pediatric preclinical testing program: model description for *in vitro* cytotoxicity testing. Pediatr Blood Cancer.

[29] Xu J, Erdreich-Epstein A, Gonzalez-Gomez I, Melendez EY, Smbatyan G, Moats RA, Rosol M, Biegel JA, Reynolds CP (2012). Novel cell lines established from pediatric brain tumors. J Neurooncol.

[30] Doan NB, Nguyen HS, Montoure A, Al-Gizawiy MM, Mueller WM, Kurpad S, Rand SD, Connelly JM, Chitambar CR, Schmainda KM, Mirza SP (2017). Acid ceramidase is a novel drug target for pediatric brain tumors. Oncotarget.

[31] Kolberg M, Strand KR, Graff P, Andersson KK (2004). Structure, function, and mechanism of ribonucleotide reductases. Biochim Biophys Acta.

[32] Kunos CA, Chiu SM, Pink J, Kinsella TJ (2009). Modulating radiation resistance by inhibiting ribonucleotide reductase in cancers with virally or mutationally silenced p53 protein. Radiat Res.

[33] Edwards LA, Woolard K, Son MJ, Li A, Lee J, Ene C, Mantey SA, Maric D, Song H, Belova G, Jensen RT, Zhang W, Fine HA (2011). Effect of brain- and tumor-derived connective tissue growth factor on glioma invasion. J Natl Cancer Inst.

[34] Wang WJ, Yao Y, Jiang LL, Hu TH, Ma JQ, Liao ZJ, Yao JT, Li DF, Wang SH, Nan KJ (2013). Knockdown of lymphoid enhancer factor 1 inhibits colon cancer progression *in vitro* and *in vivo*. PLoS One.

[35] Jawhari S, Bessette B, Hombourger S, Durand K, Lacroix A, Labrousse F, Jauberteau MO, Ratinaud MH, Verdier M (2017). Autophagy and TrkC/NT-3 signaling joined forces boost the hypoxic glioblastoma cell survival. Carcinogenesis.

[36] Fischer C, Mazzone M, Jonckx B, Carmeliet P (2008). FLT1 and its ligands VEGFB and PlGF: drug targets for anti-angiogenic therapy?. Nat Rev Cancer.

[37] Yang X, Li D, Cheng S, Fan K, Sheng L, Zhang J, Feng B, Xu Z (2014). The correlation of bone morphogenetic protein 2 with poor prognosis in glioma patients. Tumour Biol.

[38] Brigelius-Flohé R, Kipp A (2009). Glutathione peroxidases in different stages of carcinogenesis. Biochim Biophys Acta.

[39] Shimizu T, Kurozumi K, Ishida J, Ichikawa T, Date I (2016). Adhesion molecules and the extracellular matrix as drug targets for glioma. Brain Tumor Pathol.

[40] Smits M, van Rijn S, Hulleman E, Biesmans D, van Vuurden DG, Kool M, Haberler C, Aronica E, Vandertop WP, Noske DP, Würdinger T (2012). EZH2-regulated DAB2IP is a medulloblastoma tumor suppressor and a positive marker for survival. Clin Cancer Res.

[41] D’Avenia P, Porrello A, Berardo M, Angelo MD, Soddu S, Arcangeli G, Sacchi A, D’Orazi G (2006). Tp53-gene transfer induces hypersensitivity to low doses of X-rays in glioblastoma cells: a strategy to convert a radio-resistant phenotype into a radiosensitive one. Cancer Lett.

[42] Lindner I, Hemdan NY, Buchold M, Huse K, Bigl M, Oerlecke I, Ricken A, Gaunitz F, Sack U, Naumann A, Hollborn M, Thal D, Gebhardt R (2010). Alpha2-macroglobulin inhibits the malignant properties of astrocytoma cells by impeding beta-catenin signaling. Cancer Res.

[43] Soker S, Svahn CM, Neufeld G (1993). Vascular endothelial growth factor is inactivated by binding to alpha 2-macroglobulin and the binding is inhibited by heparin. J Biol Chem.

[44] Feige JJ, Negoescu A, Keramidas M, Souchelnitskiy S, Chambaz EM (1996). Alpha 2-macroglobulin: a binding protein for transforming growth factor-beta and various cytokines. Horm Res.

[45] Gettins PG, Crews BC (1994). Binding of epidermal growth factor to human alpha 2-macroglobulin. Significance for cytokine alpha 2-macroglobulin interactions. Ann N Y Acad Sci.

[46] Gettins PG, Crews BC (1993). Epidermal growth factor binding to human alpha 2-macroglobulin. Implications for alpha 2-macroglobulin-growth factor interactions. Biochemistry.

[47] Buffenstein R (2008). Negligible senescence in the longest living rodent, the naked mole-rat: insights from a successfully aging species. J Comp Physiol B.

[48] Buffenstein R (2005). The naked mole-rat: a new long-living model for human aging research. J Gerontol A Biol Sci Med Sci.

[49] Yu C, Li Y, Holmes A, Szafranski K, Faulkes CG, Coen CW, Buffenstein R, Platzer M, de Magalhães JP, Church GM (2011). RNA sequencing reveals differential expression of mitochondrial and oxidation reduction genes in the long-lived naked mole-rat when compared to mice. PLoS One.

