# Supplementary material for: Irradiation of pediatric glioblastoma cells promotes radioresistance and enhances glioma malignancy via genome-wide transcriptome changes
Source: Oncotarget. 2018 Sep 25;9(75):34122–31. doi: 10.18632/oncotarget.26137 (PMC6183347; doi:10.18632/oncotarget.26137)
Supplement: Supplementary file 1 [file oncotarget-09-34122-s001.pdf]

Irradiation of pediatric glioblastoma cells promotes radioresistance and enhances glioma malignancy *via* genome-wide transcriptome changes

SUPPLEMENTARY MATERIALS

Supplementary Table 1: Raw data of all differentially expressed genes are shown. $P < 0.05$ and experiments were performed in triplicate. See Supplementary_Table_1

Supplementary Table 2: Upregulated genes with 2-fold or higher changes are shown. $P < 0.05$ and experiments were performed in triplicate. See Supplementary_Table_2

Supplementary Table 3: Downregulated genes with 2-fold or higher changes are shown. $P < 0.05$ and experiments were performed in triplicate. See Supplementary_Table_3

Supplementary Table 4: Upregulated genes of selected enriched gene ontology categories following irradiation are shown based on sets of statistically significant changes (>2-fold changes, $P < 0.05$). See Supplementary_Table_4
