# Supplementary material for: Irradiation of pediatric glioblastoma cells promotes radioresistance and enhances glioma malignancy via genome-wide transcriptome changes
Source: Oncotarget. 2018 Sep 25;9(75):34122–31. doi: 10.18632/oncotarget.26137 (PMC6183347; doi:10.18632/oncotarget.26137)
Supplement: Supplementary file 5 [file oncotarget-09-34122-s005.docx]

**Supplementary Table 4: Upregulated genes of selected enriched gene ontology categories following irradiation are shown based on sets of statistically significant changes (>2-fold changes, *P <* 0.05)**

| **Gene ontology group** | **P-value** | Fold changes | **Gene symbol** | **Gene description** |
| --- | --- | --- | --- | --- |
| **stem cell differentiation** | **6.60E-03** | 3.637129 | **KIT** | **KIT proto-oncogene receptor tyrosine kinase** |
|  |  | 4.503667 | **GPM6A** | **glycoprotein M6A** |
|  |  | 2.094346 | **MSX1** | **msh homeobox 1** |
|  |  | 2.247623 | **MSX2** | **msh homeobox 2** |
|  |  | 11.741135 | **PDX1** | **pancreatic and duodenal homeobox 1** |
| **positive regulation of gene expression** | **7.70E-03** | 2.160103 | **CITED1** | **Cbp/p300 interacting transactivator with Glu/Asp rich carboxy-terminal domain1** |
|  |  | 3.637129 | **KIT** | **KIT proto-oncogene receptor tyrosine kinase** |
|  |  | 2.461265 | **WNT10A** | **Wnt family member 10A** |
|  |  | 2.282793 | **BMP2** | **bone morphogenetic protein 2** |
|  |  | 3.442365 | **CTGF** | **connective tissue growth factor** |
|  |  | 2.95611 | **CYP26B1** | **cytochrome P450 family 26 subfamily B member 1** |
|  |  | 2.110376 | **FGFR4** | **fibroblast growth factor receptor 4** |
|  |  | 2.602684 | **INHBA** | **inhibin beta A subunit** |
|  |  | 3.691226 | **ID1** | **inhibitor of DNA binding 1, HLH protein** |
|  |  | 3.9754 | **ID2** | **inhibitor of DNA binding 2, HLH protein** |
|  |  | 2.05908 | **LEF1** | **lymphoid enhancer binding factor 1** |
|  |  | 90.515942 | **NTRK3** | **neurotrophic receptor tyrosine kinase 3** |
|  |  | 2.334802 | **TLR4** | **toll like receptor 4** |
|  |  | 2.615161 | **TLE1** | **transducin like enhancer of split 1** |
| **positive regulation of cell proliferation** | **2.40E-02** | 2.059223 | **FOSL1** | **FOS like 1, AP-1 transcription factor subunit** |
|  |  | 2.908751 | **GRK5** | **G protein-coupled receptor kinase 5** |
|  |  | 2.527481 | **HMX2** | **H6 family homeobox 2** |
|  |  | 3.637129 | **KIT** | **KIT proto-oncogene receptor tyrosine kinase** |
|  |  | 2.020623 | **SP6** | **Sp6 transcription factor** |
|  |  | 4.558629 | **TBX3** | **T-box 3** |
|  |  | 2.442739 | **TNFRSF11A** | **TNF receptor superfamily member 11a** |
|  |  | 3.014043 | **AREG** | **amphiregulin** |
|  |  | 3.442365 | **CTGF** | **connective tissue growth factor** |
|  |  | 7.280311 | **DLL1** | **delta like canonical Notch ligand 1** |
|  |  | 2.702135 | **DPP4** | **dipeptidyl peptidase 4** |
|  |  | 42.674431 | **DYNAP** | **dynactin associated protein** |
|  |  | 5.259042 | **EDN3** | **endothelin 3** |
|  |  | 2.110376 | **FGFR4** | **fibroblast growth factor receptor 4** |
|  |  | 4.859473 | **GCNT2** | **glucosaminyl (N-acetyl) transferase 2, I-branching enzyme** |
|  |  | 2.05908 | **LEF1** | **lymphoid enhancer binding factor 1** |
|  |  | 90.515942 | **NTRK3** | **neurotrophic receptor tyrosine kinase 3** |
|  |  | 11.741135 | **PDX1** | **pancreatic and duodenal homeobox 1** |
|  |  | 2.037924 | **PGF** | **placental growth factor** |
|  |  | 2.745369 | **RAC2** | **ras-related C3 botulinum toxin substrate 2** |
|  |  | 8.168663 | **THBS1** | **thrombospondin 1** |
| **oxidation-reduction process** | **3.00E-02** | 7.083194 | **BDH1** | **3-hydroxybutyrate dehydrogenase, type 1** |
|  |  | 2.808111 | **KIAA1456** | **KIAA1456** |
|  |  | 2.282793 | **BMP2** | **bone morphogenetic protein 2** |
|  |  | 3.432834 | **CREG2** | **cellular repressor of E1A stimulated genes 2** |
|  |  | 3.124878 | **C15orf48** | **chromosome 15 open reading frame 48** |
|  |  | 2.728863 | **CRYL1** | **crystallin lambda 1** |
|  |  | 2.701761 | **CYP2J2** | **cytochrome P450 family 2 subfamily J member 2** |
|  |  | 4.409454 | **CYP26A1** | **cytochrome P450 family 26 subfamily A member 1** |
|  |  | 2.95611 | **CYP26B1** | **cytochrome P450 family 26 subfamily B member 1** |
|  |  | 2.513852 | **CYP39A1** | **cytochrome P450 family 39 subfamily A member 1** |
|  |  | 2.958365 | **CYB5R2** | **cytochrome b5 reductase 2** |
|  |  | 3.472561 | **DHRS2** | **dehydrogenase/reductase 2** |
|  |  | 2.480447 | **DCT** | **dopachrome tautomerase** |
|  |  | 3.465828 | **EGLN3** | **egl-9 family hypoxia inducible factor 3** |
|  |  | 4.012774 | **GLRX** | **glutaredoxin** |
|  |  | 7.963485 | **GPX3** | **glutathione peroxidase 3** |
|  |  | 2.328499 | **GLDC** | **glycine decarboxylase** |
|  |  | 2.153674 | **HSDL2** | **hydroxysteroid dehydrogenase like 2** |
|  |  | 2.134651 | **LOXL4** | **lysyl oxidase like 4** |
|  |  | 2.072684 | **ME3** | **malic enzyme 3** |
|  |  | 4.288283 | **PXDN** | **peroxidasin** |
|  |  | 2.457515 | **P3H2** | **prolyl 3-hydroxylase 2** |
|  |  | 2.115942 | **SARDH** | **sarcosine dehydrogenase** |
|  |  | 2.019363 | **SPR** | **sepiapterin reductase (7,8-dihydrobiopterin:NADP+ oxidoreductase)** |
|  |  | 2.424689 | **SNCA** | **synuclein alpha** |
|  |  | 3.703784 | **VAT1L** | **vesicle amine transport 1 like** |
| **sprouting angiogenesis** | **3.20E-02** | 13.747525 | **RSPO3** | **R-spondin 3** |
|  |  | 2.05908 | **LEF1** | **lymphoid enhancer binding factor 1** |
|  |  | 2.037924 | **PGF** | **placental growth factor** |
|  |  | 8.168663 | **THBS1** | **thrombospondin 1** |
| **cellular response to tumor necrosis factor** | **3.60E-02** | 2.795875 | **ADAMTS12** | **ADAM metallopeptidase with thrombospondin type 1 motif 12** |
|  |  | 3.246533 | **PPARGC1A** | **PPARG coactivator 1 alpha** |
|  |  | 5.428298 | **CHI3L1** | **chitinase 3 like 1** |
|  |  | 3.220534 | **CRHBP** | **corticotropin releasing hormone binding protein** |
|  |  | 2.023286 | **HYAL3** | **hyaluronoglucosaminidase 3** |
|  |  | 2.927765 | **NPNT** | **nephronectin** |
|  |  | 4.301679 | **KCNJ11** | **potassium voltage-gated channel subfamily J member 11** |
|  |  | 8.168663 | **THBS1** | **thrombospondin 1** |
| **inflammatory response** | **4.10E-02** | 3.637129 | **KIT** | **KIT proto-oncogene receptor tyrosine kinase** |
|  |  | 4.586202 | **TNFAIP3** | **TNF alpha induced protein 3** |
|  |  | 2.442739 | **TNFRSF11A** | **TNF receptor superfamily member 11a** |
|  |  | 4.86554 | **TNIP3** | **TNFAIP3 interacting protein 3** |
|  |  | 10.319492 | **AIM2** | **absent in melanoma 2** |
|  |  | 2.282793 | **BMP2** | **bone morphogenetic protein 2** |
|  |  | 5.428298 | **CHI3L1** | **chitinase 3 like 1** |
|  |  | 3.169818 | **C3AR1** | **complement C3a receptor 1** |
|  |  | 2.645426 | **C5AR2** | **complement component 5a receptor 2** |
|  |  | 3.220534 | **CRHBP** | **corticotropin releasing hormone binding protein** |
|  |  | 2.95611 | **CYP26B1** | **cytochrome P450 family 26 subfamily B member 1** |
|  |  | 17.941144 | **FFAR3** | **free fatty acid receptor 3** |
|  |  | 6.434821 | **GBP5** | **guanylate binding protein 5** |
|  |  | 2.023286 | **HYAL3** | **hyaluronoglucosaminidase 3** |
|  |  | 4.298401 | **KRT16** | **keratin 16** |
|  |  | 6.765047 | **TACR1** | **tachykinin receptor 1** |
|  |  | 8.168663 | **THBS1** | **thrombospondin 1** |
|  |  | 2.334802 | **TLR4** | **toll like receptor 4** |
|  |  | 2.020623 | **DUSP6** | **dual specificity phosphatase 6** |
|  |  | 9.343739 | **LUM** | **lumican** |
| **regulation of cell growth** | **7.60E-02** | 2.920671 | **FXYD2** | **FXYD domain containing ion transport regulator 2** |
|  |  | 3.522989 | **HTRA1** | **HtrA serine peptidase 1** |
|  |  | 2.422674 | **KAZALD1** | **Kazal type serine peptidase inhibitor domain 1** |
|  |  | 2.098415 | **WFDC1** | **WAP four-disulfide core domain 1** |
|  |  | 3.442365 | **CTGF** | **connective tissue growth factor** |
|  |  | 2.252145 | **SGK1** | **serum/glucocorticoid regulated kinase 1** |
| **Wnt signaling pathway** | **8.40E-02** | 2.908751 | **GRK5** | **G protein-coupled receptor kinase 5** |
|  |  | 13.747525 | **RSPO3** | **R-spondin 3** |
|  |  | 2.461265 | **WNT10A** | **Wnt family member 10A** |
|  |  | 2.213148 | **WNT7B** | **Wnt family member 7B** |
|  |  | 47.914819 | **DKK2** | **dickkopf WNT signaling pathway inhibitor 2** |
|  |  | 2.05908 | **LEF1** | **lymphoid enhancer binding factor 1** |
|  |  | 2.580052 | **PITX2** | **paired like homeodomain 2** |
|  |  | 2.695775 | **TCF7** | **transcription factor 7 (T-cell specific, HMG-box)** |
|  |  | 2.615161 | **TLE1** | **transducin like enhancer of split 1** |
|  |  | 2.164599 | **TLE4** | **transducin like enhancer of split 4** |
| **negative regulation of apoptotic process** | **8.40E-02** | 5.517826 | **BCL2A1** | **BCL2 related protein A1** |
|  |  | 4.468529 | **CD74** | **CD74 molecule** |
|  |  | 2.908751 | **GRK5** | **G protein-coupled receptor kinase 5** |
|  |  | 2.84889 | **LIMS2** | **LIM zinc finger domain containing 2** |
|  |  | 3.941924 | **NKX2-6** | **NK2 homeobox 6** |
|  |  | 4.558629 | **TBX3** | **T-box 3** |
|  |  | 3.023459 | **WT1** | **Wilms tumor 1** |
|  |  | 9.238125 | **ASCL1** | **achaete-scute family bHLH transcription factor 1** |
|  |  | 2.262002 | **BMP4** | **bone morphogenetic protein 4** |
|  |  | 3.472802 | **DHRS2** | **dehydrogenase/reductase 2** |
|  |  | 3.691226 | **ID1** | **inhibitor of DNA binding 1, HLH protein** |
|  |  | 2.05908 | **LEF1** | **lymphoid enhancer binding factor 1** |
|  |  | 2.094346 | **MSX1** | **msh homeobox 1** |
|  |  | 2.247623 | **MSX2** | **msh homeobox 2** |
|  |  | 3.529589 | **NTSR1** | **neurotensin receptor 1** |
|  |  | 2.424689 | **SNCA** | **synuclein alpha** |
|  |  | 8.168663 | **THBS1** | **thrombospondin 1** |
|  |  | 2.002775 | **UCP2** | **uncoupling protein 2** |
|  |  | 3.713038 | **VEGFB** | **vascular endothelial growth factor B** |
| **Metallopeptidase, catalytic domain** | **2.40E-02** | 3.257579 | **ADAM28** | **ADAM metallopeptidase domain 28** |
|  |  | 2.795875 | **ADAMTS12** | **ADAM metallopeptidase with thrombospondin type 1 motif 12** |
|  |  | 5.466434 | **ADAMTS14** | **ADAM metallopeptidase with thrombospondin type 1 motif 14** |
|  |  | 3.498169 | **ADAMTS5** | **ADAM metallopeptidase with thrombospondin type 1 motif 5** |
|  |  | 2.287862 | **ADAMTS9** | **ADAM metallopeptidase with thrombospondin type 1 motif 9** |
|  |  | 4.796892 | **MMP12** | **matrix metallopeptidase 12** |
|  |  | 2.583273 | **MMP17** | **matrix metallopeptidase 17** |

Experiments were performed in triplicate.
